# Effect of Cross-Linking on the Structural, Thermal, and Photocatalytic Properties of Electrospun Chitosan-Based Membranes with TiO_2_ for the Heterogeneous Photocatalysis of Dyes

**DOI:** 10.3390/molecules31213943

**Published:** 2026-11-02

**Authors:** Fabiana da Silva Lima Cardoso, Ariane Regina de Souza Rossin, Nathan José Serafini Kuhn, Josiane Caetano, Douglas Cardoso Dragunski

**Affiliations:** 1 Research Group on Electrospinning, Nanomaterials, Polymers, Electrochemistry, and Catalysis, Center of Engineering and Exact Sciences, State University of Western Paraná—UNIOESTE, Toledo 85903-000, Brazil; fabii_lima@hotmail.com (F.d.S.L.C.); ariane.rossin@hotmail.com (A.R.d.S.R.); nathan.kuhn1@unioeste.br (N.J.S.K.); 2 Department of Chemistry, State University of Maringá—UEM, Maringá 87020-900, Brazil

**Keywords:** polymer blends, photoelectrochemical degradation, AOPs

## Abstract

This study evaluated electrospun membranes based on chitosan (CTH)/polyethylene oxide (PEO) and polyvinyl alcohol (PVA)/CTH blends with titanium dioxide (TiO_2_), which were cross-linked for treatment with Reactive Red 195 dye. Characterization included SEM, FTIR, DSC, and TGA, while performance was assessed via photoelectrochemical degradation and electrocatalysis. Glutaraldehyde cross-linking was applied to CTH/PEO/TiO_2_, and citric acid with heat treatment to PVA/CTH/TiO_2_. SEM showed that CTH/PEO/TiO_2_ membranes had partial cross-linking and poor wet stability, whereas PVA/CTH/TiO_2_ exhibited satisfactory stability. FTIR confirmed structural changes in organic groups after cross-linking. DSC revealed reduced melting points for PEO and PVA, indicating cross-linking. TGA demonstrated improved thermal stability for both fiber types. In electrochemical tests, the cross-linked chitosan electrodes were non-conductive and unsuitable for photoelectrochemical degradation. The non-cross-linked electrode achieved 60% dye removal, but adsorption contributed significantly, so results could not be attributed solely to degradation. In photodegradation assays, crosslinked CTH/PEO/TiO_2_ membranes adsorbed 75% of the dye before light exposure and showed only 22% additional removal under irradiation. Non-crosslinked PVA/CTH/TiO_2_ membranes dissolved in the medium, making the tests invalid. Overall, cross-linking enhanced stability and adsorption but limited electrochemical applicability, highlighting the need to balance structural integrity and functional performance in membrane-based dye treatment systems.

## 1. Introduction

The growing pollution that threatens natural resources, such as potable water, requires society to adopt sustainable consumption and/or treatment alternatives to prevent the depletion of this resource [1]. Among emerging technological alternatives, Advanced Oxidation Processes (AOPs) have gained prominence for their ability to completely degrade dyes into less harmful by-products. Within this group, heterogeneous photocatalysis has been studied due to its high efficiency in degrading persistent organic compounds—a result of hydroxyl radical formation induced by the irradiation of a semiconductor, such as titanium dioxide (TiO_2_), which exhibits excellent oxidizing power and the ability to promote the complete mineralization of pollutants [2,3].

However, the efficiency of this technique—widely used in photocatalytic studies—can be limited by factors such as the recombination of photogenerated charges (e^−^/h^+^), a crucial step in hydroxyl radical formation, and the difficulty of separating the semiconductor when used in suspension in aqueous reaction media [4]. To overcome this limitation, a promising strategy is to immobilize TiO_2_ onto electrospun polymer membranes. The electrospinning technique enables the production of nanofibers with high surface area and porosity, thereby facilitating the interaction between contaminants and the photocatalyst. This type of support enhances the adsorption of pollutants (e.g., dyes) and reduces the agglomeration of incorporated nanoparticles, thereby improving the material’s performance [5,6].

Water contamination by dyes is a concerning environmental challenge, primarily caused by improper disposal of industrial effluents from the textile, paper, and plastics industries. These compounds exhibit high chemical stability and intense coloration, which hinder sunlight penetration and impact the aquatic ecosystem [7].

Various polymers can be used to produce electrospun polymeric membranes, including chitosan (CHT), polyethylene oxide (PEO), and polyvinyl alcohol (PVA). Chitosan is a biopolymer derived from chitin that possesses biodegradable and biocompatible properties, as well as functional groups that facilitate the adsorption of pollutants [8]. PEO is a hydrophilic polymer that improves membrane solubility and processability. At the same time, PVA is known to be biodegradable, biocompatible, non-toxic, and water-soluble [8,9]. Chemical crosslinking and heat treatment are employed to enhance the stability of polymeric membranes in aqueous media. Some cross-linking agents used include glutaraldehyde (GLA) and citric acid (CA) [10,11,12].

Considering this, the study aimed to develop photocatalytic electrospun polymer membranes composed of chitosan, polyethylene oxide, and polyvinyl alcohol, incorporating the semiconductor titanium dioxide, to evaluate the potential of these materials for degrading the dye Reactive Red 195 via heterogeneous photocatalysis in an aqueous medium.

## 2. Results

**Figure 1:**
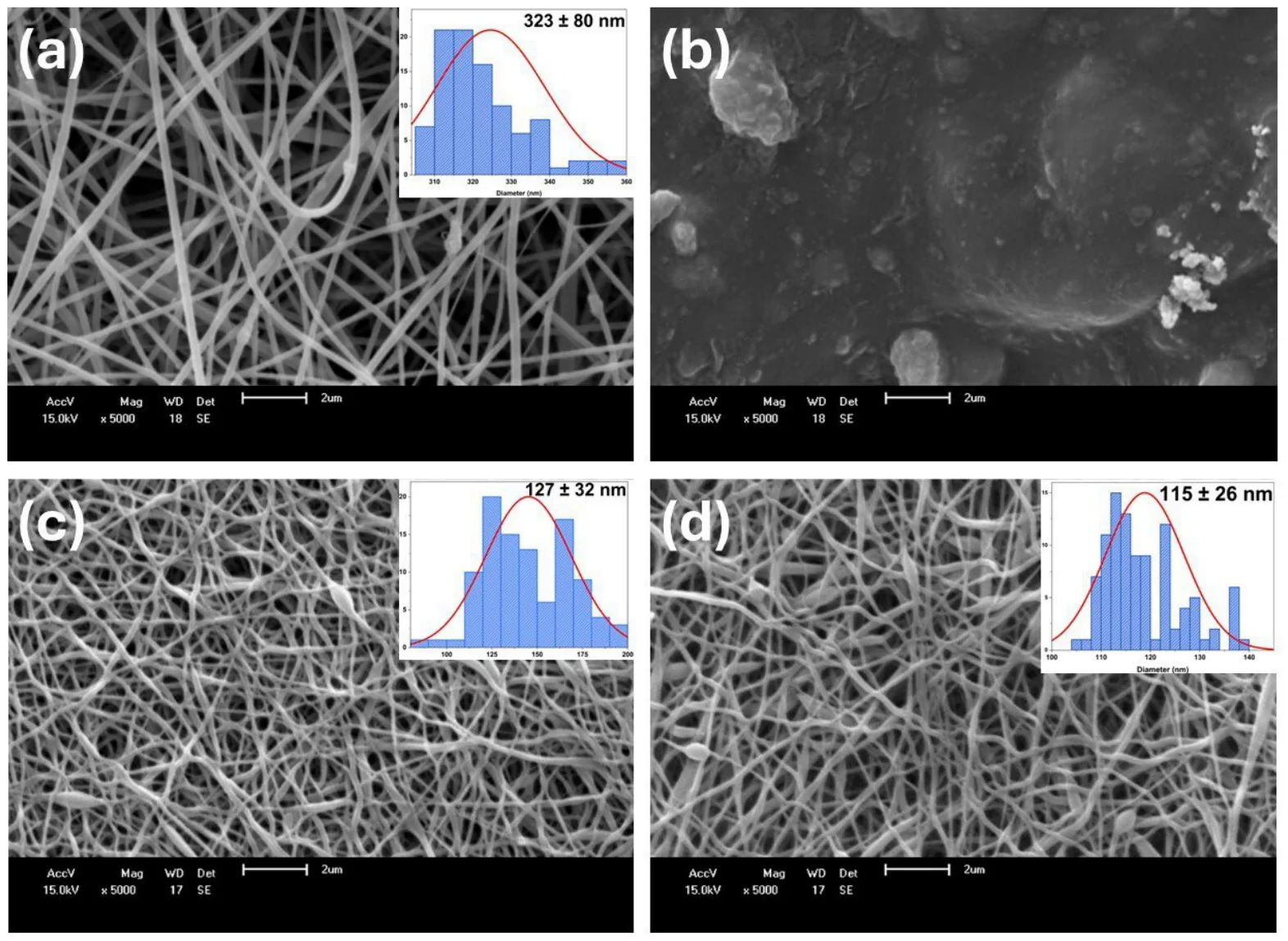
Scanning Electron Microscopy images (**a**) Non-crosslinked and (**b**) crosslinked CTH/PEO/TiO_2_ membranes, and (**c**) non-crosslinked and (**d**) crosslinked PVA/CTH/TiO_2_ membranes. The histograms show the fiber diameter distribution, and the red lines represent the Gaussian fitting curves. Optical photographs of the membranes are provided in Figure S1 (Supplementary Material).

After electrospinning CTH/PEO polymer membranes, crosslinking studies using glutaraldehyde (GLA) immersion were conducted to enhance the material’s resistance to aqueous environments. To this end, GLA solutions at concentrations of 1% and 10% were prepared. Initial crosslinking studies showed that these concentrations were too high, yielding a brittle material that did not meet the requirements for use in aqueous media. Consequently, the decision was made to reduce the GLA concentration to 0.1%. Figure 1 illustrates the morphology of the CTH/PEO/TiO_2_ and PVA/CTH/TiO_2_ membranes before and after the crosslinking process. Before crosslinking, the CTH/PEO/TiO_2_ membranes were composed of smooth fibers with an average diameter of 323 ± 80 nm (Figure 1a), along with randomly distributed beads. Following immersion in GLA, the membranes lost their fibrous appearance, indicating that crosslinking was unsatisfactory (Figure 1b). Neto et al. [13] highlight that the CTH cross-linking process depends on temperature, glutaraldehyde concentration, and degree of deacetylation, among other factors. The most common method, as described by Sencadas et al. [10]. It consists of vaporizing 50% *v*/*v* GLA for at least 8 h at room temperature. Schiffman and Schauer [14] cross-linked chitosan membranes by directly adding a Schiff base to a solution containing medium-molecular-weight chitosan, thereby carrying out the cross-linking in a single step.

The PVA/CTH blend has already been reported in the literature as having good electrospinnability. Çay et al. [15] evaluated different PVA/CTH blend ratios for electrospinning, obtaining a maximum average diameter of 300 nm for the 50/50 polymer mixture. For PVA/CTH/TiO_2_, the membranes prior to crosslinking (Figure 1c) had a diameter of 127 ± 32 nm. After crosslinking, the electrospun membranes exhibited fibrous characteristics, with a diameter of 115 ± 26 nm (Figure 1d). Nasikhudin et al. [16] observed a decrease in the diameter of electrospun PVA/CTH fibers (8/2 ratio) upon the addition of 2% TiO_2_, without a crosslinking process. The authors note that the average diameter decreased due to a reduction in the viscosity of the PVA/TiO_2_ mixture upon the addition of CTH. In another study. Nasikhudin et al. [17] evaluated the effect of crosslinking PVA/CTH/TiO_2_ via heat treatment and immersion in 2.5% GLA in acetone/ethanol. The authors observed that the GLA crosslinking process was effective, and the fibers remained stable after 20 days of immersion in water. This study is the first report in the literature on the crosslinking of electrospun membranes with citric acid.

**Figure 2:**
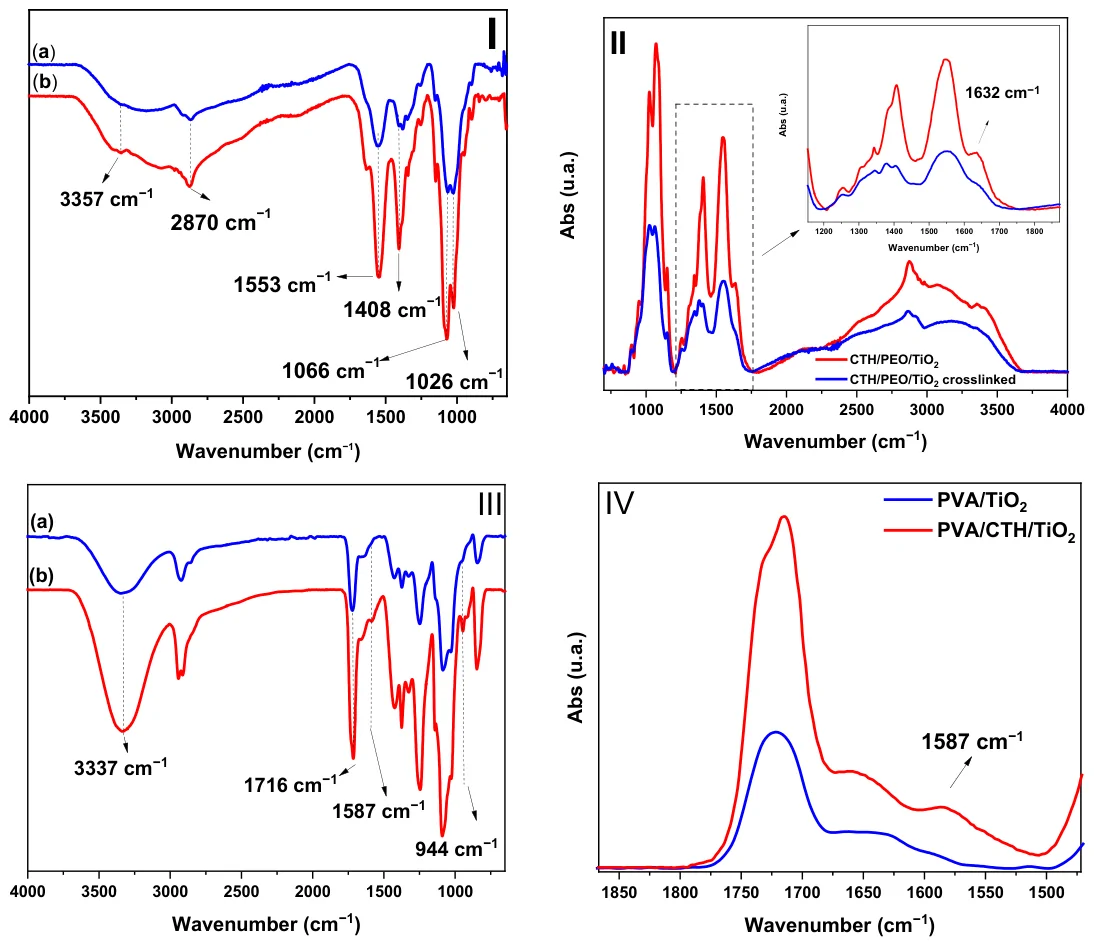
(**I**) FTIR-ATR of (a) crosslinked and (b) non-crosslinked CHT/PEO/TiO_2_ membranes. (**II**)—Absorption spectrum for CHT/PEO/TiO_2_. (**III**)—FTIR-ATR of (a) crosslinked PVA/TiO_2_ and (b) crosslinked PVA/CHT/TiO_2_ membranes. (**IV**)—Absorption spectra for crosslinked PVA/TiO_2_ and PVA/CHT/TiO_2_. To enhance spectral comparison, the spectra has been vertically offset along the transmittance axis.

The FTIR and absorption spectra of the CTH/PEO/TiO_2_ membranes are shown in Figure 2I,II. For the non-crosslinked membrane (Figure 2I-a), a broad band is observed at 3357 cm^−1^, attributed to inter- and intramolecular bonds involving O-H and N-H groups from chitosan and the O-H group from PEO [14]. The peaks at 2870 and 1408 cm^−1^ correspond to C-H bonds in methylene (CH_2_) groups. Characteristic bands for the chitosan amine group are observed at 1553 cm^−1^ and 1066 cm^−1^ (aliphatic amines). Finally, the characteristic PEO band at 1026 cm^−1^ corresponds to the vibrational stretching of the ether group (C-O-C) [18]. In the crosslinked membrane (Figure 2I-b), a decrease in band intensity is evident. The crosslinking process in CTH/PEO/TiO_2_ is accompanied by reduced intensities of bands associated with CH_2_, amide, aldehyde, and amino groups, as shown in Figure 2. In the cross-linked membrane (Figure 2II), a decrease in band intensity is observed, particularly in the region associated with amine groups (~1550–1590 cm^−1^), indicating a reduction in the availability of chitosan primary amino groups (-NH_2_) during the reaction with GLA. Cross-linking between GLA and CTH occurs through the formation of Schiff bases (imine bonds) between aldehyde and amino groups. However, the band at 1632 cm^−1^, which overlaps with the chitosan amide I band, should be interpreted with caution. The reduced intensity of the N–H bending bands (~1550–1590 cm^−1^) provides stronger evidence of cross-linking, as described by Kildeeva et al. [19].

FTIR and absorption spectra of PVA/CTH/TiO_2_ membranes were compared to those of crosslinked PVA/TiO_2_ to identify stretching vibrations associated with CTH; these are shown in Figure 2III,IV. Compared to PVA/TiO_2_, PVA/CTH/TiO_2_ exhibits several similar stretching bands, including the broad band at 3337 cm^−1^ (associated with O-H stretching) and the band at 1716 cm^−1^. The band at 1716 cm^−1^ is consistent with the formation of ester bonds resulting from the reaction between citric acid and the hydroxyl groups of PVA [20]. However, this spectral region may contain overlapping contributions from unreacted residual citric acid (which also absorbs near 1716 cm^−1^) and residual acetate groups from the partially hydrolyzed PVA [21]. Therefore, although the band at 1716 cm^−1^ is consistent with cross-linking, its interpretation requires caution. At 1587 cm^−1^ (Figure 2IV), the appearance of a stretching band associated with CTH N-H vibrations is observed, indicating hydrogen bonding interactions between PVA and CTH. The peak at 944 cm^−1^ is attributed to C-O single bond stretching vibrations from the CTH. These results are consistent with those reported for PVA/CTH [20,21] and for PVA/CTH crosslinked with GLA [22]. No data on the electrospinning of citric acid-crosslinked PVA/CTH/TiO_2_ has been reported in the literature.

It is important to note that while FTIR spectroscopy provides valuable information about structural changes in the membranes, the interpretation of bands in the carbonyl region (1600–1750 cm^−1^) requires caution due to potential overlap from different functional groups, including ester, carboxylic acid, and acetate groups. Therefore, the evidence for crosslinking obtained by FTIR should be considered together with the complementary data from TGA and DSC, which further support the occurrence of crosslinking in both membrane systems.

**Figure 3:**
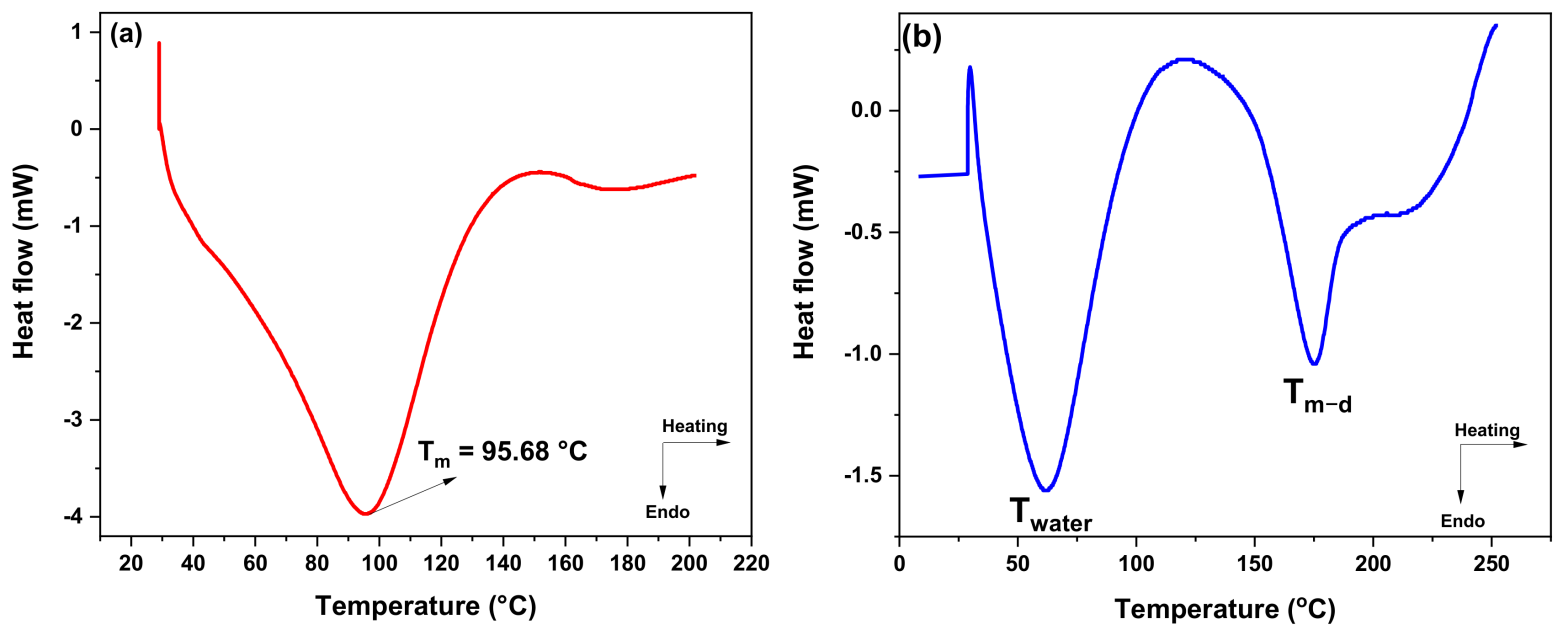
Differential Scanning Calorimetry analysis of electrospun membranes of (**a**) CTH/PEO/TiO_2_ and (**b**) PVA/CTH/TiO_2_.

The literature reports that for CTH/PEO blends at an 80/20 ratio, the DSC curves show an endothermic melting peak (T_m_) at 54 °C and an endothermic evaporation peak (T_water_) at 99.8 °C. For crosslinked CTH/PEO/TiO_2_ (Figure 3a), the PEO melting peak disappears. This disappearance may be related to decreased PEO crystallinity and increased free PEO resulting from crosslinking [23]. Furthermore, a decrease in T_water_ to 94.8 °C is noticeable. Sun et al. [24] explain that the cross-linking process reduces the amino groups—and consequently the hydrogen bonds between CHT and water—thereby facilitating their release.

Data for PVA/CTH reported in the literature shows only thermal events. The first event involves water molecules—both strongly and weakly bound within the PVA/CTH structure—that can be released at a peak temperature (T_water_) of 150 °C. Although DSC experiments were conducted at temperatures above 0 °C to characterize the thermal transitions of the polymers, the literature indicates that analysis of the sub-zero region can provide valuable information on the water states present in hydrophilic membranes [25]. The presence of different water states—free water, freezable bound water, and non-freezable water—is directly related to the interaction between the polymer matrix (PVA/PEO) and the solvent and is influenced by the blend composition and the crosslinking density of the system [21]. The second thermal event corresponds to the simultaneous melting and degradation (T_m-d_) of the PVA, with a peak temperature of 190 °C [22]. For the crosslinked PVA/CTH/TiO_2_ membrane (Figure 3b), decreases in T_water_ and T_m-d_ to 61 °C and 174 °C, respectively, were observed. This can be explained as follows: the decrease in T_water_ indicates reduced interaction between H_2_O molecules and the PVA/CTH structure, resulting from the formation of a crosslinked network with citric acid. Furthermore, crosslinking reduces the number of intra- and intermolecular bonds in PVA, thereby decreasing T_m-d_ [11].

**Figure 4:**
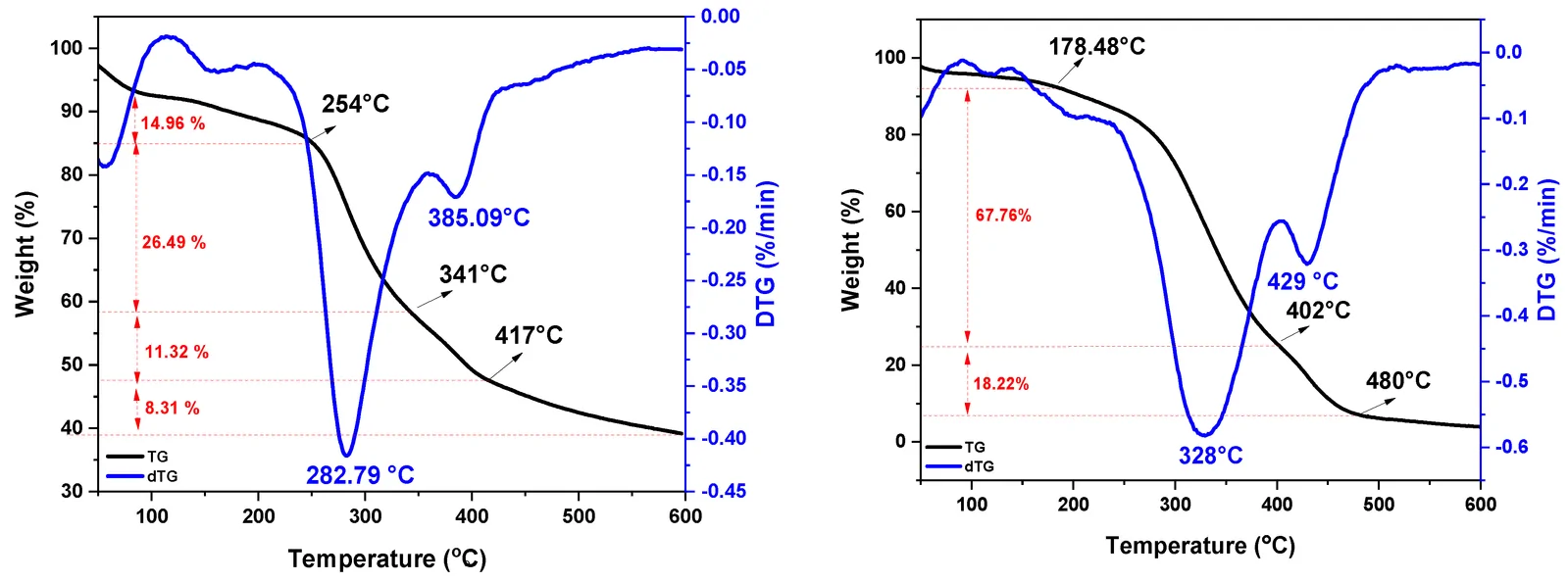
Thermogravimetric (TG) curves and derivative degradation curves (dTG) of PEO/CTH/TiO_2_ and PVA/CTH/TiO_2_ membranes.

For CTH, as reported in the literature, a thermal event associated with adsorbed water is observed up to 130 °C, followed by a more prominent event peaking at 300 °C, corresponding to the degradation and depolymerization of chitosan. For PEO, a single thermal event associated with its degradation is reported, peaking at 390 °C [26]. For the CTH/PEO/TiO_2_ mixture (Figure 4), three thermal events are observed: the first attributed to water loss, the second—showing the highest percentage of mass loss—related to CTH degradation, and the third to PEO degradation. The maximum mass loss for CTH/PEO/TiO_2_ was approximately 42%, indicating high thermal stability resulting from hydrogen bonding between the CTH functional groups and the TiO_2_. Hussein et al. [27] observed similar behavior, noting that the thermal stability of CTH/TiO_2_ membranes tends to increase with rising TiO_2_ concentration.

Three degradation stages are reported for PVA/CHT. The first is associated with water loss at temperatures near 100 °C. The second stage arises from the cleavage of chitosan rings and the decomposition reactions of other functional groups, peaking at 295 °C. The final stage corresponds to the decomposition of the residue, which peaks at 368 °C [15]. A shift in the degradation peaks is observed for the crosslinked membranes containing TiO_2_. Rossin et al. [12] and Fraga et al. [11] have reported that crosslinking improves the thermal stability of PVA membranes. Furthermore, studies indicate that adding TiO_2_ to PVA/CHT membranes enhances stability [28]. Therefore, the increased thermal stability of the PVA/CHT/TiO_2_ materials studied here may result from a synergistic effect involving both crosslinking and the presence of TiO_2_.

### 2.1. Photoelectrochemical Degradation Study

**Figure 5:**
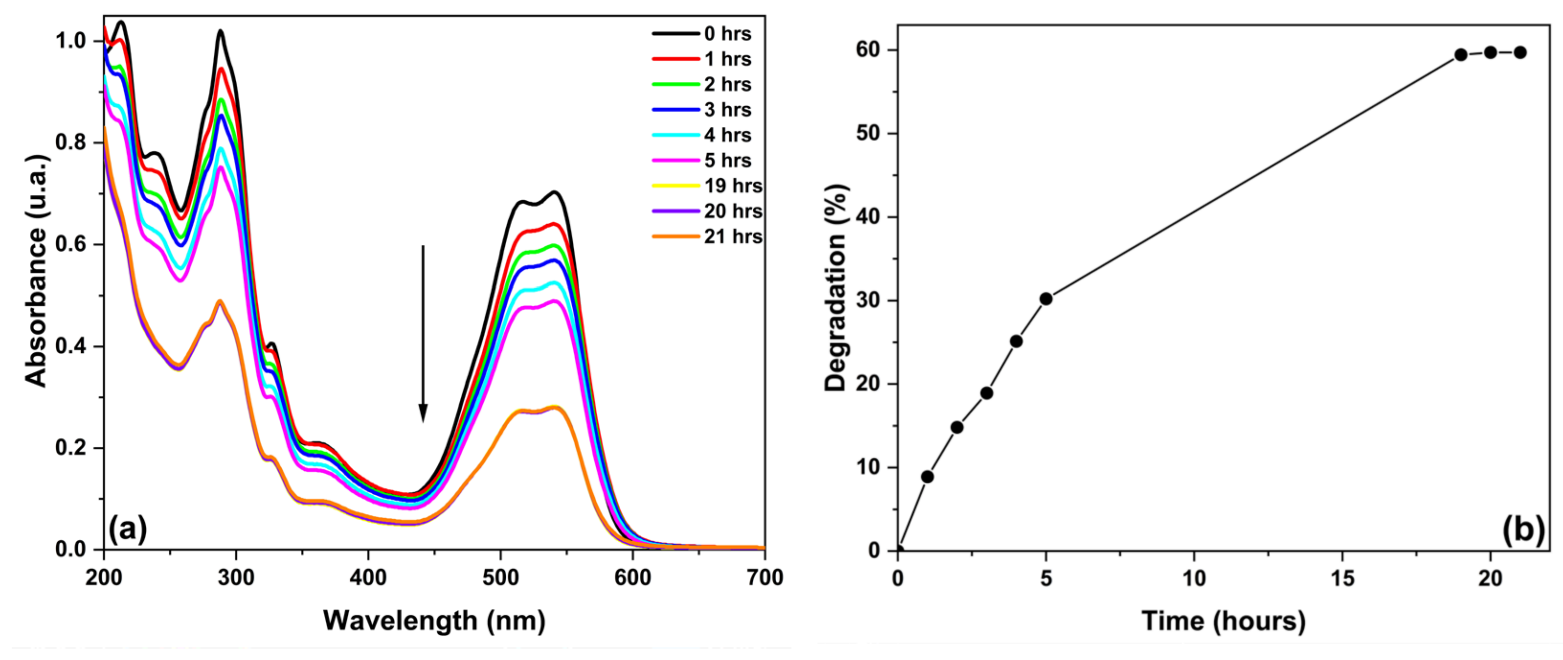
Plot of (**a**) absorbance measurements and (**b**) degradation percentage, both versus time, for the CTH/PEO/TiO_2_ membrane.

Photoelectrochemical degradation was performed using both the non-crosslinked and crosslinked electrodes. The photocurrent density of the non-crosslinked electrode decreased rapidly from 1.6 to 0.5 µA cm^−2^ within the first 4 h of operation, then stabilized at a very low value. This pronounced decrease is indicative of severe surface deactivation, which can be attributed to: (i) the loss of structural integrity of the non-crosslinked polymeric matrix, leading to detachment of the photoactive layer; (ii) the accumulation of degradation byproducts on the electrode surface, which block active sites and promote charge recombination; and/or (iii) photocorrosion of the semiconductor under prolonged illumination. The stabilization at 0.5 µA cm^−2^ after 4 h suggests that while some residual photoelectrochemical activity persists, the electrode is largely deactivated for practical applications. This behavior contrasts with that of crosslinked electrodes, which typically exhibit greater stability under similar conditions. Figure 5 presents the absorbance measurements and the degradation over time. Observing the graph in Figure 5b at a fixed wavelength of 541 nm—corresponding to the chromophore group of the Reactive Red 195 dye molecule—it can be inferred that degradation reached approximately 60% over a period of about 19 h.

However, no degradation or adsorption was observed on the electrode. However, no significant degradation or adsorption was observed for this electrode. Figure S2 (Supplementary Material) shows the electrode before and after the photoelectrocatalytic degradation study, highlighting the intense dye coloration, indicating its persistence in solution. Turkten and Cinar [29] report that the dye Reactive Red 195 (RR195) absorbs onto the TiO_2_ surface via sulfonic and carbonyl groups in its chemical structure. Using Density Functional Theory (DFT), the same authors demonstrated that the decolorization of RR195 is primarily associated with cleavage of the azo bond (-N=N-), the chromophore responsible for the dye’s color. This cleavage can generate aromatic intermediates, such as naphthalene and benzene, which, in subsequent reactions, can be oxidized to low-molecular-weight carboxylic acids—such as oxalic and acetic acids—during more advanced mineralization processes. Therefore, the lack of decolorization observed in our experiments indicates that the photoelectrode did not even effectively promote the initial step of azo bond cleavage under the tested conditions.

The cross-linking process rendered the electrode non-conductive. Consequently, an attempt was made to calcine the electrode, but all the material deposited on it was lost during the process. Due to the detachment of part of the electrospun membrane from the electrode edges and unsuccessful attempts to crosslink the membrane and improve its adhesion to the FTO surface, the material was deemed unsuitable for photoelectrochemical catalysis applications.

### 2.2. Photocatalysis Studies

**Figure 6:**
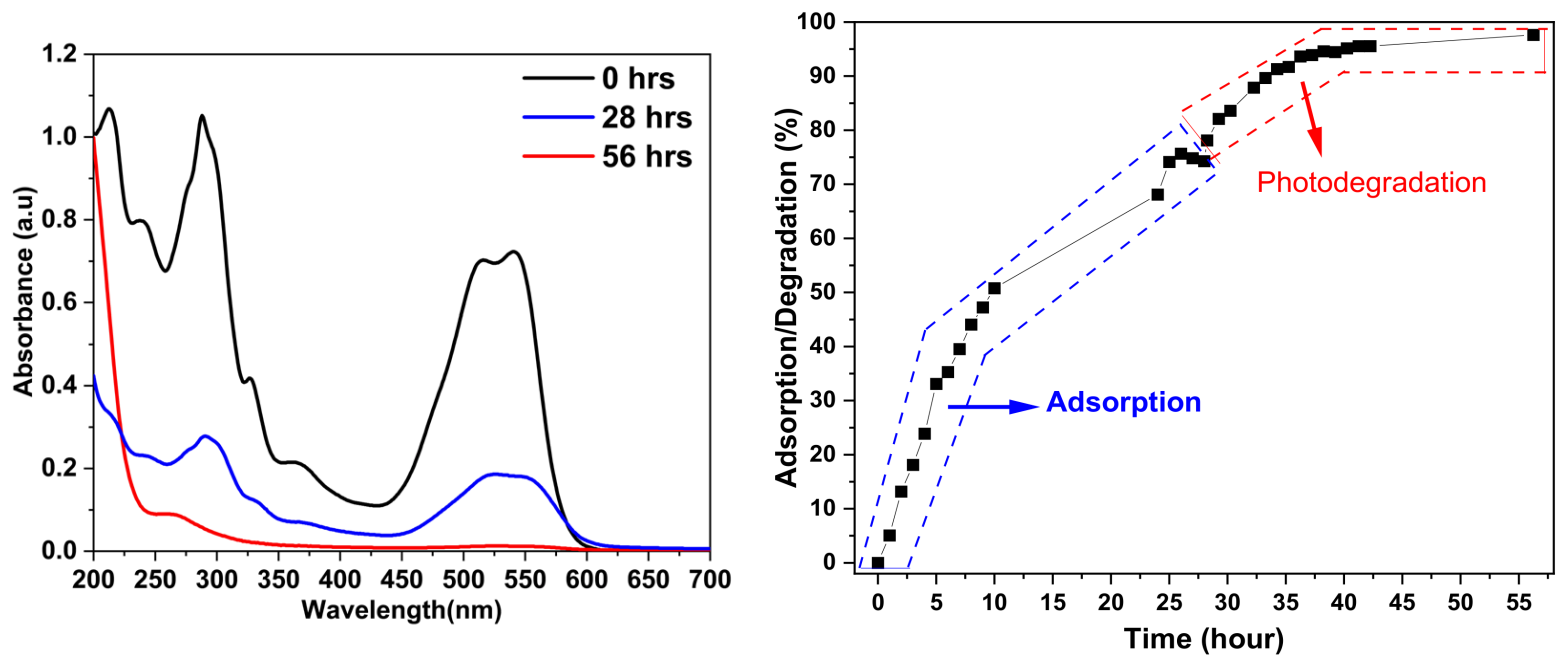
Photodegradation spectra with the cross-linked CTH/PEO/TiO_2_ membrane and adsorption/degradation over time.

Both the non-crosslinked and crosslinked membranes were tested in photocatalysis studies, with the results shown in Figure 6. The TiO_2_ employed in this study exhibits an anatase-dominated crystalline structure, known for its high photocatalytic activity due to efficient charge separation at the anatase/rutile phase boundary [30]. Furthermore, the intrinsic hydrophilicity of the TiO_2_ surface—which can be further enhanced by UV-induced photo-induced hydrophilicity—favors the adsorption of water molecules and the Reactive Red 195 dye onto the photocatalyst surface, promoting the initial steps of the heterogeneous photocatalytic process [31].

Initially, an equilibration period was conducted, during which the photocatalytic material was kept shielded from light. The non-crosslinked membrane failed to withstand the aqueous medium and began to disintegrate during the equilibration phase; consequently, the study could not be completed. Regarding the GLA-crosslinked CHT/PEO/TiO_2_ membrane, the dye absorbance did not stabilize during the equilibration period; therefore, measurements were continued for an extended period. This period lasted 28 h. Once equilibrium was reached, the light was turned on, and the process continued for another 28 h. Figure 5 presents the absorbance plots at the start of the experiment, after adsorption, after photodegradation, and the plot showing adsorption/degradation over time.

It was observed that approximately 75% of the dye was removed from solution by adsorption onto the membrane during the initial 28 h of dark equilibration. This value was considered the membrane’s maximum adsorption capacity under the experimental conditions, as the absorbance remained constant for three consecutive hours. After the adsorption equilibrium was reached, irradiation was initiated, and a further decrease in dye concentration was observed. During the subsequent 28 h irradiation period, an additional 22 percentage points of dye removal were achieved, reducing the residual dye concentration from 25% to approximately 3% of its initial value. Therefore, the photocatalytic stage contributed to the further removal of the dye beyond that achieved by adsorption alone, resulting in an overall dye removal efficiency of approximately 97%. These results demonstrate that the combination of adsorption and photocatalysis is highly effective in removing Reactive Red 195 from aqueous solutions.

Furthermore, an attempt was made to electrospin a solution without TiO_2_ to determine how much dye could be removed in its absence; however, the resulting fibers were too thin to withstand cross-linking. Additionally, although electrospinning was feasible after adding titanium dioxide, the yield of electrospun material was not satisfactory from a cost-effectiveness perspective. Moreover, since part of the result relied on adsorption—which requires a secondary treatment step and thus deviates from the study’s primary goal of achieving a single-step process—it was deemed worthwhile to modify the polymer composition to improve the electrospun material.

**Figure 7:**
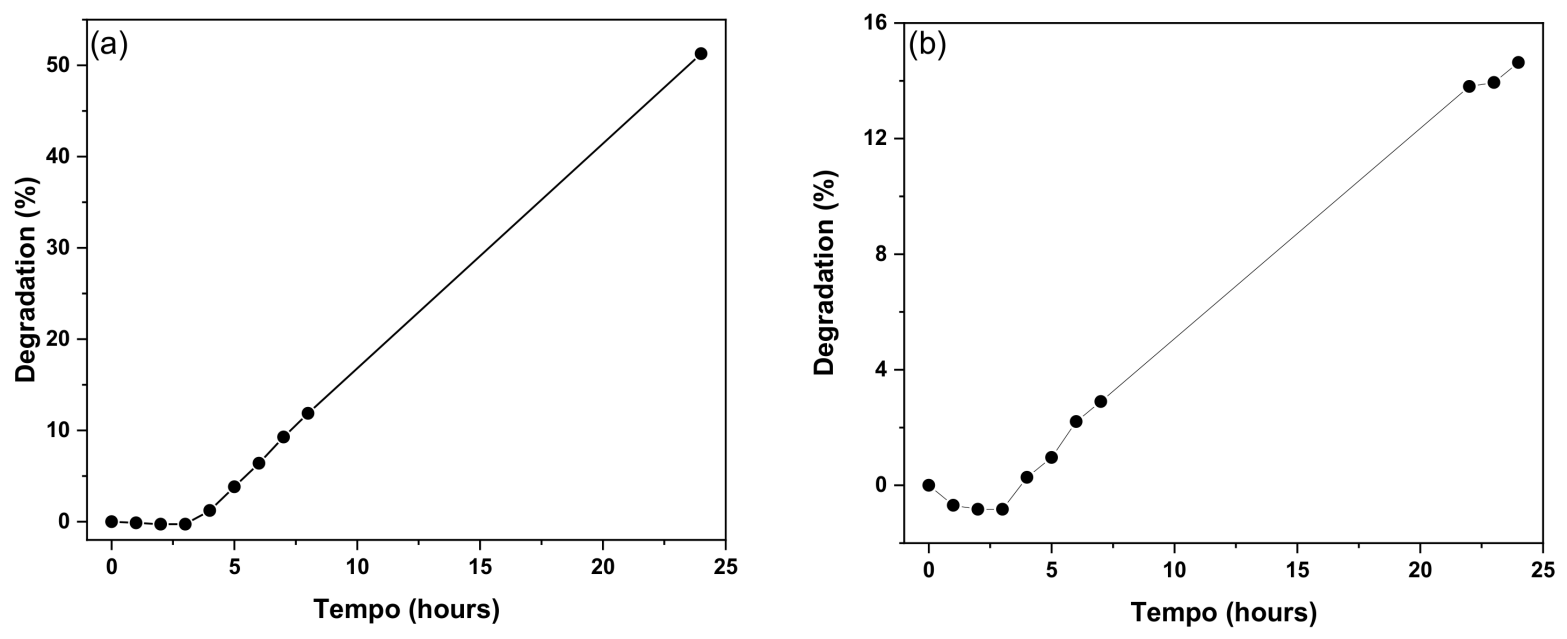
Degradation study of the dye Reactive Red 195 (35 mg/L and 30 mL) using (**a**) PVA/CTH/TiO_2_ and (**b**) CTH/PVA polymeric membranes.

To this end, the decision was made to replace PEO with PVA in the polymer membrane composition. Figure 7 presents the results of the degradation study using electrospun PVA/CTH/TiO_2_ and PVA/CTH polymer membranes.

For the PVA/CHT/TiO_2_ polymer membrane (Figure 7a), a steady and continuous increase in dye degradation is observed after the equilibration period (in the absence of light), reaching approximately 51% after 1440 min of study. This indicates that the produced material possesses photocatalytic potential. Compared to the study shown in Figure 6 regarding the electrospun CHT/PEO/TiO_2_ material, the adsorption process here is significantly lower, suggesting that the decrease in RR195 dye concentration is primarily due to photodegradation. For the PVA/CHT polymer membrane (Figure 7b), the results highlight the importance of TiO_2_ for dye degradation efficiency. The absence of TiO_2_ resulted in only a 15% drop in dye concentration. However, in this case, it cannot be stated that the result was due to photocatalytic degradation, as neither chitosan nor PVA has the capacity to induce the formation of reactive species under light irradiation; thus, this result—albeit modest—was likely influenced by the adsorption capacity of chitosan, owing to the presence of amine (-NH_2_) and hydroxyl (-OH) functional groups. These results indicate that altering the polymer composition affects not only the material’s stability but also the dye-membrane surface interaction, thereby influencing the efficiency of the photocatalytic process.

## 3. Materials and Methods

### 3.1. Materials

Chitosan (CTH, 85% deacetylated; Mw = 87 kDa) was acquired from Golden-Shell Biochemical. The semiconductors PEO (Mw = 65.000 g mol^−1^) and titanium dioxide (TiO_2_, purity 99.5%, particle size < 100 nm and mixture of rutile and anatase phases) were purchased from Sigma-Aldrich. Glacial acetic acid (99.8% *v*/*v*) was purchased from Neon. Water was purified by reverse osmosis.

### 3.2. Preparation of the Polymer Solution

The solution was initially prepared with a fixed mass of CTH/PEO at 3% (*w*/*v*). A 90/10 ratio of CTH/PEO was dissolved in 10 mL of a 90% (*v*/*v*) acetic acid/water solution. After complete dissolution, TiO_2_ equivalent to 10% of the CTH/PEO mass was added to the mixture, and the mixture was then stirred magnetically for 24 h. This solution was designated as CTH/PEO/TiO_2_.

For the preparation of CTH, PVA, and TiO_2_, a 2% (*w*/*v*) CTH solution was first prepared by dissolving CTH in a 1% (*v*/*v*) acetic acid solution in distilled water. The PVA solution was prepared as described by Rossin et al. [12]. After cooling, citric acid was added at 15% (*w*/*w*) relative to the PVA mass, and the mixture was stirred until fully dissolved. Subsequently, a solution was prepared at a 70/30 ratio by combining the PVA/citric acid and chitosan solutions. Finally, titanium dioxide (TiO_2_) was added at 10% (*w*/*w*) relative to the total polymer mass, and the mixture was stirred magnetically. This solution was designated PVA/CTH/TiO_2_.

### 3.3. Electrospinning Parameters

The solutions were electrospun using a system comprising a high-voltage power supply, a syringe pump (connected to the negative terminal), a grounded static collector (connected to the positive terminal), and a 10 mL glass syringe with a metal needle (1.5 mm in diameter). The optimal conditions used to produce the CTH/PEO/TiO_2_ fibers were an applied voltage of 13 kV and a needle-to-collector distance of 12 cm. Electrospinning was performed at room temperature (T ≈ 21.5 ± 3.5 °C), with a flow rate of 1 mL/h and an air humidity of approximately 42.5% ± 12.5%. For cross-linking, the fibers were immersed in 0.05% glutaraldehyde for 1 min, then dried at room temperature.

For PVA/CTH/TiO_2_, the optimal conditions used for fiber production were an applied voltage of 24 kV and a needle-to-collector distance of 12 cm. Electrospinning was performed at room temperature (T ≈ 21.5 ± 3.5 °C) using a flow rate of 0.4 mL/h and an air humidity of approximately 42.5% ± 12.5%. For cross-linking, the fibers were kept in an oven at 120 °C for 2 h.

### 3.4. Characterization of the Fibers

The morphological characteristics of the fibers were observed using Scanning Electron Microscopy (SEM). Fiber images were captured using a SHIMADZU SUPERSCAN SS-550 (Shimadzu Corporation, Kyoto, Japan). Samples were mounted on double-sided carbon tape and sputter-coated with a thin layer of gold (30 nm thick) using a BAL-TEC SCD 050 (BAL-TEC, Balzers, Liechtenstein) sample coater. From the SEM images obtained, the diameters of randomly selected fibers (n = 100) were measured using ImageJ (v. 2.0.0-rc-3). Mean diameters were reported as mean ± standard deviation (SD).

To evaluate the structural characteristics of the fibers, Fourier Transform Infrared (FTIR) spectral analysis was performed using the Attenuated Total Reflectance (ATR) module. The analysis was conducted at 25 °C over the 650–4000 cm−1 range at a resolution of 1 cm^−1^ using a PerkinElmer FTIR spectrometer (PerkinElmer, Inc., Waltham, MA, USA).

To evaluate the thermal stability of the components after fiber production, differential scanning calorimetry (DSC) was performed using a Shimadzu DSC-60 (Shimadzu Corporation, Kyoto, Japan) thermal analyzer. Experimental conditions included a nitrogen atmosphere at 40 mL/min and a heating rate of 10 °C/min, with no cooling. Heating was performed over the temperature range of 30–400 °C.

Thermogravimetric analysis (TGA) was used to evaluate the thermal stability of the PVP/VA powder and the nanofiber. A PerkinElmer STA 6000 (PerkinElmer, Inc., Waltham, Massachusetts, USA) thermal analyzer was used in a nitrogen atmosphere at a flow rate of 40 mL min^−1^ and a heating rate of 10 °C min^−1^ over the range of 30 °C to 600 °C.

### 3.5. Photoelectrode Fabrication

The photoelectrode was prepared using the electrospinning parameters described above and a 1 × 2 cm semiconductor plate. The plate was fixed to the static collector of the electrospinning system, and a 1 cm^2^ area was covered with the electrospun membranes. For photoelectrochemical measurements, the semiconductor plate with the electrospun membrane was used as the working electrode. The electrode was fully immersed in the electrolyte, and the membrane’s entire surface area (1 cm^2^) was illuminated. Photocurrent densities were calculated by dividing the measured current by the geometric area of the membrane (A = 1 cm^2^): J = I/A.

### 3.6. Photocatalysis

The study was conducted in a photoreactor equipped with a 300 W Osram Ultra-Vitalux UV-A/UV-B xenon lamp (OSRAM GmbH, Munich, Germany; measured intensity: 46 W/m^2^) for 24 h; the distance between the lamp and the solution was 28.5 cm, and the system temperature was maintained at 21.0 °C using a thermostatic bath. The absorbance of the dye solution (35 mg L^−1^) was measured using a Shimadzu-1800 UV-Vis (Shimadzu Corporation, Kyoto, Japan) spectrophotometer over the 200–700 nm spectral range.

### 3.7. Photoelectrochemical Degradation

The photoelectrochemical degradation activity of the produced electrode was evaluated in a reaction medium containing 35 mg/L of a dye. Na_2_SO_4_ at 0.1 mol/L was used as the supporting electrolyte. Measurements were performed using a 10 W LED lamp (λ = 365 nm) positioned at 2 cm from the CTH/PEO/TiO_2_ photoelectrode, providing an estimated light intensity of ~159 mW cm^−2^ at the electrode surface. In addition to the working electrode, the electrochemical cell—a single-compartment setup—comprised a platinum counter electrode and an Ag/AgCl (3 mol/L KCl) reference electrode. Absorbance measurements were used to monitor dye degradation efficiency with a Shimadzu UV-Vis spectrophotometer (1800) (Shimadzu Corporation, Kyoto, Japan) over 200–700 nm throughout the degradation study.

## 4. Conclusions

Characterization of the CTH/PEO/TiO_2_ electrospun membranes demonstrates that immersion in GLA solution causes partial cross-linking, since the aqueous stability of the fibers after the cross-linking process was not maintained. The PVA/CTH/TiO_2_ membranes cross-linked with citric acid and heated to a temperature showed satisfactory fiber stability in an aqueous medium. Fourier Transform Infrared Spectroscopy (FTIR) demonstrated structural changes in the organic groups for CTH/PEO/TiO_2_ and PVA/CTH/TiO_2_ membrane crosslinking, although the degree of crosslinking observed for CTH/PEO/TiO_2_ was considered unfavorable.

Differential scanning calorimetry (DSC) analysis showed crosslinking in both membranes, as evidenced by the displacement of the PEO and PVA melting peaks to lower temperatures, corroborating the structural modification promoted by the chemical treatment, as demonstrated by FTIR. Additionally, the TGA indicated increased thermal stability for the CTH/PEO/TiO_2_ and PVA/CTH/TiO_2_ fibers, reinforcing the influence of the crosslinking process on the materials’ thermal resistance.

In the Photoelectrochemical degradation study, electrochemical analysis showed that the cross-linked CTH electrodes did not conduct electrical current, rendering their application in photoelectrochemical degradation unfeasible. The non-cross-linked electrode showed 60% degradation; however, the results indicate that the model dye adsorbed onto the produced electrode. This observation indicates that the results cannot be entirely attributed to photoelectrode degradation.

For the CTH/PEO/TiO_2_ membranes, crosslinking with glutaraldehyde provided partial stability in aqueous media. In photodegradation tests, the crosslinked CTH/PEO/TiO_2_ membranes adsorbed 75% of the dye before light exposure, with an additional 22% removal under irradiation. In contrast, non-crosslinked PVA/CTH/TiO_2_ membranes were unsuitable for reliable testing due to their high solubility in aqueous media. When purchasing the PVA/CTH and PVA/CTH/TiO_2_ membranes, it was noted that approximately 50% of the dye degradation is due to TiO_2_, as membranes without TiO_2_ show degradation below 15%.

## Data Availability

The original contributions presented in this study are included in the article/Supplementary Material. Further inquiries can be directed to the corresponding authors.

## Supplementary Materials

The following supporting information can be downloaded at: https://www.mdpi.com/article/10.3390/molecules31213943/s1, Figure S1: Optical photographs of the membranes (a) non-crosslinked and (b) crosslinked CTH/PEO/TiO_2_ membranes, and (c) non-crosslinked and (d) crosslinked PVA/CTH/TiO_2_ membranes; Figure S2: Optical photographs of the CTH/PEO/TiO_2_ photoelectrode (a) before and (b) after the photoelectrochemical degradation study.
