# Supplementary material for: Effect of Cross-Linking on the Structural, Thermal, and Photocatalytic Properties of Electrospun Chitosan-Based Membranes with TiO2 for the Heterogeneous Photocatalysis of Dyes
Source: Molecules. 2026 Nov 2;31(21):3943. doi: 10.3390/molecules31213943 (PMC13764717; doi:10.3390/molecules31213943)
Supplement: Supplementary file 1 [file molecules-31-03943-s001.zip › molecules-4500699-supplementary.pdf]

Effect of cross-linking on the structural, thermal, and photocatalytic properties of electrospun Chitosan-Based Membranes with TiO_2 for the heterogeneous photocatalysis of dyes.

Fabiana da Silva Lima Cardoso[1,2], Ariane Regina de Souza Rossin[1], Nathan José Serafini Kuhn[1], Josiane Caetano[1], Douglas Cardoso Dragunski[1*]

Supplementary Material

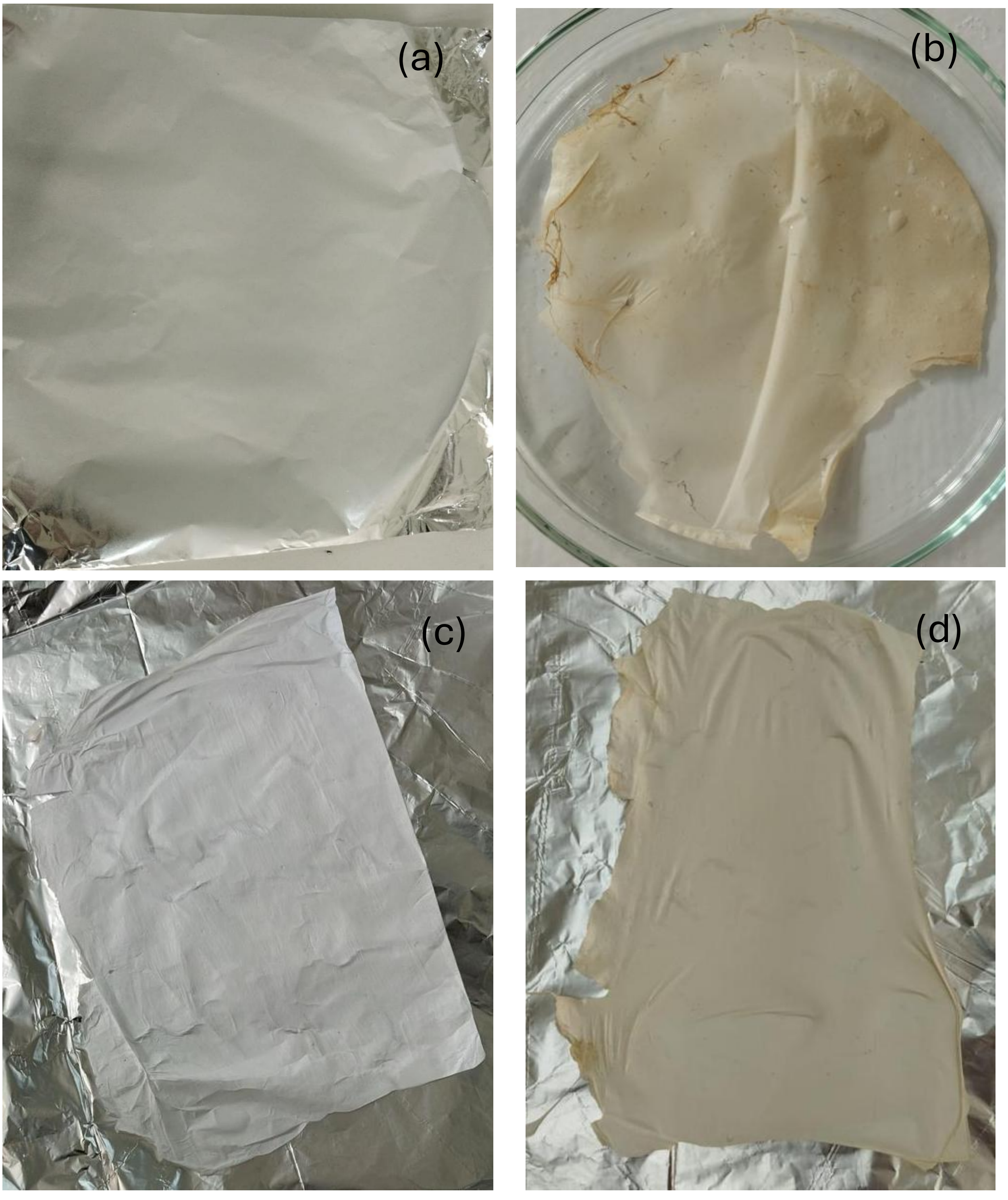

Figure S1. Optical photographs of the membranes(a) Non-crosslinked and (b) crosslinked CTH/PEO/TiO_2 membranes, and (c) non-crosslinked and (d) crosslinked PVA/CTH/TiO_2 membranes.

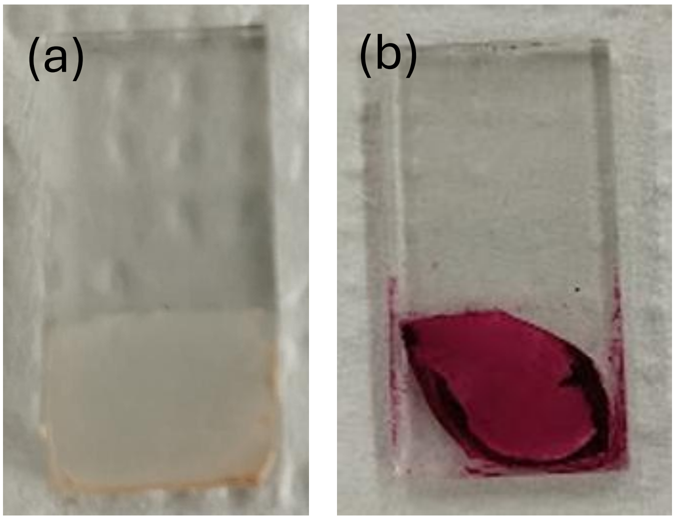

Figure S2. Optical photographs of the CTH/PEO/TiO_2 photoelectrode (a) before and (b) after the photoelectrochemical degradation study
